# CNS-PNETs with *C19MC* amplification and/or LIN28 expression comprise a distinct histogenetic diagnostic and therapeutic entity

**DOI:** 10.1007/s00401-014-1291-1

**Published:** 2014-05-20

**Authors:** Tara Spence, Patrick Sin-Chan, Daniel Picard, Mark Barszczyk, Katharina Hoss, Mei Lu, Seung-Ki Kim, Young-Shin Ra, Hideo Nakamura, Jason Fangusaro, Eugene Hwang, Erin Kiehna, Helen Toledano, Yin Wang, Qing Shi, Donna Johnston, Jean Michaud, Milena La Spina, Anna Maria Buccoliero, Dariusz Adamek, Sandra Camelo-Piragua, V. Peter Collins, Chris Jones, Nabil Kabbara, Nawaf Jurdi, Pascale Varlet, Arie Perry, David Scharnhorst, Xing Fan, Karin M. Muraszko, Charles G. Eberhart, Ho-Keung Ng, Sridharan Gururangan, Timothy Van Meter, Marc Remke, Lucie Lafay-Cousin, Jennifer A. Chan, Nongnuch Sirachainan, Scott L. Pomeroy, Steven C. Clifford, Amar Gajjar, Mary Shago, William Halliday, Michael D. Taylor, Richard Grundy, Ching C. Lau, Joanna Phillips, Eric Bouffet, Peter B. Dirks, Cynthia E. Hawkins, Annie Huang

**Affiliations:** 1 Division of Hematology-Oncology, Department of Pediatrics, The Hospital for Sick Children, Arthur and Sonia Labatt Brain Tumor Research Centre, Peter Gilgan CRL,686 Bay Street, 17th Floor, 179712, Toronto, ON M5G0A4 Canada; 2 Department of Laboratory Medicine and Pathobiology, University of Toronto, Toronto, ON Canada; 3 Department of Pathology, The Hospital for Sick Children, Toronto, ON Canada; 4 Department of Neurosurgery, Seoul National University Children’s Hospital, Seoul, South Korea; 5 Department of Neurosurgery, Asan Medical Center, Seoul, South Korea; 6 Department of Neurosurgery, Kumamoto University, Kumamoto, Japan; 7 Division of Pediatric Hematology/Oncology and Stem Cell Transplantation, Children’s Memorial Hospital, Chicago, IL USA; 8 Center for Cancer and Blood Disorders, Children’s National Medical Center, Washington, DC USA; 9 Department of Neurosurgery, Children’s Hospital of Los Angeles, Los Angeles, CA USA; 10 Oncology Department, Schneider Hospital, Petach Tikva, Israel; 11 Department of Neuropathology Huashan Hospital, Fudan University, Shanghai, China; 12 Department of Pathology, Shanghai Children’s Hospital Affiliated Shanghai Jiao Tong University School of Medicine, Shanghai, China; 13 Department of Pediatrics, Children’s Hospital of Eastern Ontario, Ottawa, ON Canada; 14 Department of Pathology and Laboratory Medicine, Children’s Hospital of Eastern Ontario, Ottawa, ON Canada; 15 Paediatric Haematology and Oncology Division, University of Catania, Sicily, Italy; 16 Pathology Unit Meyer Children’s Hospital, Florence, Italy; 17 Department of Pathomorphology, Jagiellonian University Medical College, Krakow, Poland; 18 Department of Pathology, University of Michigan, Ann Arbor, MI USA; 19 Department of Pathology, University of Cambridge, Cambridge, UK; 20 Department of Paediatric Molecular Pathology, Institute of Cancer Research, Sutton, UK; 21 Division of Pediatric Hematology Oncology, Rafic Hariri University Hospital, Beirut, Lebanon; 22 Department of Pathology and Laboratory Medicine, American University of Beirut Medical Center, Beirut, Lebanon; 23 Medical and Department of Neuropathology, Sainte-Anne Hospital, University Paris V Descartes, Paris, France; 24 Department of Pathology and Laboratory Medicine, University of California, San Francisco, CA USA; 25 Department of Pathology, Children’s Hospital Central California, Madera, CA USA; 26 Department of Neurosurgery, University of Michigan Medical School, Ann Arbor, MI USA; 27 Division of Pathology, John Hopkins University School of Medicine, Baltimore, MD USA; 28 Department of Anatomical and Cellular Physiology, Chinese University of Hong Kong, Hong Kong, China; 29 Department of Pediatrics, Duke University School of Medicine, Durham, NC USA; 30 Department of Pediatrics, Virginia Commonwealth University, Richmond, VA USA; 31 Division of Neurosurgery, Arthur and Sonia Labatt Brain Tumor Research Centre, The Hospital for Sick Children, Toronto, ON Canada; 32 Department of Pediatric Oncology, Alberta Children’s Hospital, Calgary, AB Canada; 33 Department of Pathology and Laboratory Medicine, University of Calgary, Calgary, AB Canada; 34 Departments of Pediatrics, Faculty of Medicine, Ramathibodi Hospital, Bangkok, Thailand; 35 Department of Neurology, Children’s Hospital Boston, Boston, MA USA; 36 Northern Institute for Cancer Research, Newcastle University, Newcastle Upon Tyne, UK; 37 Neuro-oncology Division, St Jude Children’s Research Hospital, Memphis, TN USA; 38 Department of Pediatric Laboratory Medicine, The Hospital for Sick Children, Toronto, ON Canada; 39 Children’s Brain Tumor Research Centre, Queen’s Medical Centre University of Nottingham, Nottingham, UK; 40 Texas Children’s Cancer Center, Baylor College of Medicine, Houston, TX USA

## Abstract

Amplification of the *C19MC* oncogenic miRNA cluster and high LIN28 expression has been linked to a distinctly aggressive group of cerebral CNS-PNETs (group 1 CNS-PNETs) arising in young children. In this study, we sought to evaluate the diagnostic specificity of *C19MC* and LIN28, and the clinical and biological spectra of *C19MC* amplified and/or LIN28+ CNS-PNETs. We interrogated 450 pediatric brain tumors using FISH and IHC analyses and demonstrate that *C19MC* alteration is restricted to a sub-group of CNS-PNETs with high LIN28 expression; however, LIN28 immunopositivity was not exclusive to CNS-PNETs but was also detected in a proportion of other malignant pediatric brain tumors including rhabdoid brain tumors and malignant gliomas. *C19MC* amplified/LIN28+ group 1 CNS-PNETs arose predominantly in children <4 years old; a majority arose in the cerebrum but 24 % (13/54) of tumors had extra-cerebral origins. Notably, group 1 CNS-PNETs encompassed several histologic classes including embryonal tumor with abundant neuropil and true rosettes (ETANTR), medulloepithelioma, ependymoblastoma and CNS-PNETs with variable differentiation. Strikingly, gene expression and methylation profiling analyses revealed a common molecular signature enriched for primitive neural features, high LIN28/LIN28B and DNMT3B expression for all group 1 CNS-PNETs regardless of location or tumor histology. Our collective findings suggest that current known histologic categories of CNS-PNETs which include ETANTRs, medulloepitheliomas, ependymoblastomas in various CNS locations, comprise a common molecular and diagnostic entity and identify inhibitors of the LIN28/let7/PI3K/mTOR axis and DNMT3B as promising therapeutics for this distinct histogenetic entity.

**Electronic supplementary material:**

The online version of this article (doi:10.1007/s00401-014-1291-1) contains supplementary material, which is available to authorized users.

## Introduction

Primitive neuroectodermal tumors of the central nervous system (CNS-PNET) are a heterogeneous group of pediatric neoplasms composed of poorly differentiated neuroepithelial cells with varying degrees of divergent neural, astrocytic and ependymal differentiation. According to the current WHO CNS tumor working classification, CNS-PNETs are grouped into several histologic categories: CNS neuroblastoma/ganglioneuroblastoma, medulloepithelioma (MEP), ependymoblastoma (EPB) and classical CNS-PNET (PNET-NOS) [14]. In 2000, Eberhart et al. [4] described a new CNS-PNET variant arising primarily in infancy, which displayed histological features of both neuroblastoma and EPB, and were distinguished by the presence of true and pseudo-rosettes on a background of abundant neuropil. These tumors, termed ‘embryonal tumors with abundant neuropil and true rosettes’ (ETANTRs), correlated with very poor patient prognosis with a mortality rate of 76 % and a median survival of 9 months [1, 5, 21].

Li et al. [13] first reported *C19MC* amplification was enriched in cerebral CNS-PNETs with variant histologic features including tumors called ETANTR, MEP, EPB and PNETs with atypical features, thus suggesting that these conventional histologic sub-classes may represent closely related molecular entities. Indeed, Korshunov et al. [10] reported *C19MC* amplification in 37/40 tumors with a histologic diagnosis of ETANTR or EPB. In addition, *C19MC* amplification has been reported in tumors with mixed features of ETANTR and MEP [2, 16]. Subsequent studies demonstrated that up-regulation of the RNA-binding pluripotency gene, *LIN28* [11, 17], correlated closely with *C19MC* amplification thus suggesting that LIN28 may represent an attractive immuno-diagnostic marker for this distinct molecular sub-group of cerebral CNS-PNETs. However, the relative diagnostic significance of *C19MC* and LIN28, and the molecular and therapeutic relationship of these different histologic sub-classes of CNS-PNETs remain to be completely elucidated.

To identify relevant therapeutic pathways for these tumors, we sought in this study to first evaluate the diagnostic specificity of *C19MC* amplification and LIN28 expression for CNS-PNETs, and define the histopathologic and clinical features of CNS-PNETs with *C19MC* amplification and/or LIN28 expression. We compared global gene expression and methylation data from *C19MC* amplified and/or LIN28+ CNS-PNETs with various histologic diagnostic labels and anatomic locations, and investigated pharmacologic inhibitors of LIN28/let-7/mTOR signaling and DNMT3B on growth of a novel cell line derived from a non-*C19MC* amplified group 1 CNS-PNET.

## Materials and methods

### Tumor and nucleic acid samples

Tumor specimens and clinical information were collected with consent as per protocols approved by Hospital Research Ethics Board at participating institutions. A total of 450 primary pediatric brain tumors with various histologic diagnoses—103 CNS-PNETs, 45 atypical rhabdoid teratoid tumors (ATRTs), 128 medulloblastomas (MBs), 105 ependymomas (EPNs), 50 high-grade gliomas (HGGs) and 20 choroid plexus carcinomas (CPCs) were examined in this study (Supplemental Table 1). All ATRTs diagnoses were confirmed for genetic alterations of *SMARCB1/INI1* by Multiplex Ligation mediated PCR and/or targeted gene sequencing and for loss of SMARCB1/INI1/BAF47 protein expression by immunostaining. Of the 54 group 1 CNS-PNETs examined for detailed histopathologic correlates (Table 1 and Supplemental Table 2), 33 were previously reported [13, 17]; and 21 additional tumors with a diagnosis of CNS-PNET, ETANTR or embryonal tumor with multilayered rosettes (ETMR), MEP and EPB were collected for this study. Three tumors with an initial diagnosis of EPB were histologically reviewed and re-classified as ETANTR (PNET 5, 39 and 54). Tumor DNA and RNA were extracted by standard methods, quantified using NanoDrop Analyzer, and analyzed using the Illumina HT-12 v4 gene expression and 450 K Methylation arrays and OmniQuad 2.5 M SNP genotyping arrays (http://www.illumina.com) to generate gene expression, methylation, and copy number profiles. Methylation profiling was performed at The Centre for Innovation at Genome Quebec; all other analyses were performed at The Centre of Applied Genomics (TCAG) at the Hospital for Sick Children.

**Table 1:** Histopathologic and clinical features of *C19MC* amplified/LIN28+ CNS-PNETs

	Total	Histologic categories			
		ETANTR/EPB	MEP	PNET^a^	PNET^b^
	54	22	12	11	9
C19MC status^c,d^					
Number analyzed	51	21	12	9	9
Amplified/overexpressed	43 (84 %)	20 (95 %)	9 (75 %)	7 (78 %)	7 (78 %)
Not amplified/overexpressed	8 (16 %)	1 (5 %)	3 (25 %)	2 (22 %)	2 (22 %)
LIN28 IHC^c,d^					
Number analyzed	40	20	10	10	0
Positive	40	20	10	10	0
C19MC/LIN28 status^c^					
Number analyzed	37	19	10	8	0
C19MC not amplified/LIN28+	5 (14 %)	1 (5 %)	2 (20 %)	2 (25 %)	0
Location^c^					
Number analyzed	54	22	12	11	9
Cerebral hemispheres	41 (76 %)	14 (62 %)	8 (67 %)	10 (91 %)	9 (100 %)
Non-cerebral	13 (24 %)	8 (38 %)	4 (33 %)	1 (9 %)	0 (0 %)
Gender^c^					
Number analyzed	53	22	12	10	9
Male:female	24:29	12:10	6:6	4:6	2:7
Age at diagnosis (months)^e^					
Number analyzed	52	22	12	11	7
Median (range)	29 (0.5–180)	29 (7–60)	23 (0.5–54)	34 (10–107)	32 (18–180)
Metastatic status^c^					
Number analyzed	37	13	10	10	4
M0:M+	24:13	8:5	6:4	7:3	3:1
Treatment^c^					
Number analyzed	41	18	11	9	3
Chemotherapy only	20 (49 %)	11 (61 %)	3 (27 %)	5 (56 %)	1 (33 %)
Chemotherapy and Radiation^f^	11 (27 %)	4 (22 %)	4 (36 %)	2 (22 %)	1 (33 %)
None	10 (24 %)	3 (17 %)	4 (36 %)	2 (22 %)	1 (33 %)
Survival status^c^					
Number analyzed	46	17	12	11	6
Status (alive:dead)	10:36	4:13	4:8	1:10	1:5
Survival (months)					
Histologic categories^g^					
Number analyzed	36	15	11	10	n/a
Median survival ± SD (95 % CI)	13 ± 2.0 (9.0–17.0)	10 ± 2.5 (5.1–15.0)	13 ± 6.2 (0.9–25.1)	19 ± 8.6 (2.2–35.8)	n/a
Treatment^g^		Treated	Untreated		
Number analyzed	36	30	6		
Median survival ± SD (95 % CI)	12 ± 1.9 (8.3–15.7)	0.1 ± 1.8 (0.0–3.7)	13 ± 1.9 (9.3–16.7)		

*ETANTR* embryonal tumor with abundant neuropil and true rosettes, *EPB* ependymoblastoma, *MEP* medulloepithelioma, *PNET* primitive neuroectodermal tumor, *M0* no metastasis, *M+* M1–M3 metastasis as per Chang criteria

^a^ Includes PNET with ependymal and anaplastic features

^b^ Only institutional pathology diagnosis available

^c^ Pearson Chi-Square

^d^ Subset of tumors analyzed on TMAs (including 5/50 for C19MC FISH and 7/40 for LIN28 IHC)

^e^ Kruskal–Wallis Test

^f^ Includes one patient treated with radiation only

^g^ Log-Rank (Mantel–Cox) test

### Histology, immunohistochemistry and fluorescence in situ hybridization

Hematoxylin and eosin (H and E) staining was performed using standard protocols. Fluorescence in situ hybridization (FISH) and immunohistochemistry (IHC) were performed on 5-μm formalin-fixed paraffin-embedded (FFPE) sections for individual tumors or tissue microarrays (TMA). TMA construction was as previously detailed [18]. Tumor tissue blocks and corresponding slides were reviewed by an experienced neuropathologist (CH) for diagnostic accuracy and adequacy of tissues; samples that had extensive necrosis or <60 % tumor content were discarded. Representative tumor areas were identified, and three 1-mm tissue cores were selected with the goal of obtaining a sampling accuracy >95 % for each tumor represented on the TMA [6, 8]. A variety of tissues including liver, fetal cerebellum, placenta, breast carcinoma, and basal cell carcinoma were included on each array to serve as internal controls for various immuno-stains.

For FISH analyses, pre-labeled BAC probes (http://www.tcag.ca/cytogenomics) mapping to chr19q13.42 (RP11-381E3: 162, 225 bp) and an internal control chr19p13.11 locus (RP11-451E20: 165,783 bp) were used as described previously [13]. For IHC analyses, a heat-induced antigen retrieval process was used, followed by blocking endogenous peroxidase and biotin. Primary anti-LIN28 (#3978) and anti-phospho-S6 (Ser240/244) (#5364) antibodies were purchased from Cell Signaling Technology (Boston, MA, USA) and anti-DNMT3B (ab13604) was purchased from Abcam (Toronto, ON, CA). Antibody reactivity was visualized using the VectaStain ABC detection kit, Vector Laboratories (Burlingame, CA, USA).

For each protein, cytoplasmic and/or nuclear immunopositivity was visually scored based on both strength (0, 1, 2, 3–3+) and distribution (<25, 25–50, 51–75, and >75 % of tumor cells). Only tumors with strong staining (3–3+) in >25 % of tumor cells were considered to be positive. Tumors with FFPE slides were scored based on analyses of the whole tumor section, while tumors on TMAs were assessed on the basis of staining patterns in at least two of three tissue cores. Human testicular germ cell tumor tissue was used as a positive control for LIN28 staining, while sections processed in parallel without primary antibodies were used as negative controls. All immunohistochemical stains were scored independently by TS, DP, MB and NH, and reviewed by AH and CH.

### Gene expression and methylation array data analyses

Gene expression data from 59 primary CNS-PNETs arising in different locations were generated using the Illumina HT-12v4 arrays and normalized as previously described [17]. Methylation data generated using the Illumina human 450 k arrays for 45 primary CNS-PNETs were background-normalized in Genome Studio (v. 2011.1) to obtain beta values for downstream analyses. All X and Y chromosome probes (*n* = 11,649), single-nucleotide polymorphisms (dbSNP, *n* = 88,679), and unannotated probes (relative to hg19, *n* = 65) were excluded leaving a total of 385,184 probes for methylation analyses. Genes and probes were ranked by largest coefficient of variation and standard deviation, respectively. Unsupervised hierarchical clustering (HCL; Partek Genomics Suite, v6.6) of gene expression and methylation data were, respectively, established using Pearson’s Dissimilarity performed iteratively on 200–2,000 genes and 200–10,000 probes to identify a minimal gene set associated with the most stable gene expression and methylation tumor cluster patterns (Supplemental Fig. 2).

### Statistical analyses

Median age at diagnosis was compared using Kruskal–Wallis test and Log Rank (Mantel–Cox) analysis was used to assess survival. All other clinical and biological characteristics were compared using Pearson Chi-Square (*χ* ^2^). A *p* value of <0.05 was regarded as significant for all analyses. All statistical analyses were done using SPSS version 22.0.

### Cell culture and growth assays

To generate stable tumor cell line, A664, a pre-treatment primary tumor from a non-*C19MC* amplified/LIN28+ CNS-PNET (PNET398) was dissociated by gentle manual trituration followed by passage through a 40-μm mesh filter. Cells were grown in low-adhesion tissue culture flasks (Sarstedt, Montreal, QC, CA) in defined serum-free media at 37 °C, 5 % CO_2_. Culture media consisted of human neural stem cell proliferation media (Stem Cell Technologies, Vancouver, BC, CA) supplemented with heparin, epidermal growth factor (EGF, 10 ng/ml, Sigma Aldrich, St. Louis, MO, USA), and fibroblast growth factor (FGF, 10 ng/ml, Stem Cell Technologies, Vancouver, BC, CA). Cells were dissociated using Accumax (Millipore, MA, USA) by manual trituration and re-plated at ~40 % confluency (20,000 cells/ml) and maintained stably for >10 consecutive passages.

 For drug treatment and growth assays, A664 cells were plated in 96-well dishes at 2,000 cells/well and incubated overnight. Cells were then treated with rapamycin (Sigma Aldrich, St. Louis, MO, USA), 5-azacytidine (Sigma Aldrich, St. Louis, MO, USA) or vorinostat (Selleckchem, Burlington, ON, CA) and evaluated for changes in cell proliferation assays at various days post-treatment using CellTiter 96^®^ AQueous One Solution Cell Proliferation Assay kit (Promega, Madison WI, USA). Viable cell numbers were determined based on absorbance at 575 nm using a Versamax microplate reader (Molecular Devices, Sunnyvale, CA, USA).

### Quantitative real-time (qRT-PCR), western blot and siRNA knockdown analyses

For LIN28 knockdown, A664 cells were plated at a density of 1.5 × 10^5^ cells/ml in 6-well plates. Scrambled control or LIN28-specific siRNA (Fisher Scientific, Ottawa, ON, CA) was transiently transfected into cells using Lipofectamine 2000 (Life Technologies, Burlington, ON, CA) as per the manufacturer’s protocol. Cells were harvested at 48-h post-transfection for protein extraction and analysis by Western blotting. Western blot analysis and chemiluminescence detection were performed using standard protocols. Secondary HRP-linked donkey anti-Rabbit or HRP-linked sheep anti-Mouse (#NAV934, 931) was from GE Healthcare (Baie-d’Urfe, QC, CA).

For miRNA-specific qRT-PCR, single-stranded cDNA was synthesized from 10 ng of RNA using a miR-specific stem-loop reverse-transcription RT-primer and the TaqMan^®^ MicroRNA Reverse Transcription Kit (Life Technologies, Burlington, ON, CA). qRT-PCR was performed using TaqMan^®^ Universal PCR Master Mix, no AmpErase^®^ UNG (Life Technologies, Burlington, ON, CA) according to the manufacturer’s instructions; miRNA expression levels were normalized relative to RNU6B using the Δ*C*_t_ method, and compared to that of 16-week-old whole human fetal brain control.

## Results

### CNS-PNETs with *C19MC* amplification and LIN28 immunopositivity span a histologic and anatomic spectrum of tumors

We [13, 17] and others [10, 11] previously reported frequent amplification of the *C19MC* oncogenic miRNA cluster and high LIN28 protein expression in an aggressive sub-group of CNS-PNETs. To comprehensively define the clinico-pathologic features of these newly recognized molecular entities, we first tested the diagnostic robustness of the *C19MC* and LIN28 markers in a spectrum of pediatric brain tumors for which materials were available for central re-review of histopathology and confirmation of histologic diagnosis according to the current WHO CNS tumor classification criteria. These comprised an institutional cohort of 128 MBs, 45 ATRTs, 105 EPNs, 50 HGGs, 20 CPCs, and 103 CNS-PNETs from various institutions; 450 and 416 samples had materials available for FISH and LIN28 IHC analyses. As reported previously, *C19MC* amplification or copy number gains were restricted to and detected in 24.3 % (25/103) of CNS-PNETs, while LIN28 immunopositivity was observed in 21.4 % (22/103) of all CNS-PNETs. However, in contrast to *C19MC* amplification, cytoplasmic LIN28 immunopositivity was not restricted to CNS-PNETs but was also observed in a spectrum of other tumors including 24.4 % (11/45) of ATRTs and 19.5 % (8/41) of HGGs (Supplemental Table 1**)**. These results indicate that LIN28 immunopositivity alone may not be sufficient to establish a diagnosis of CNS-PNETs.

**Fig. 1:**
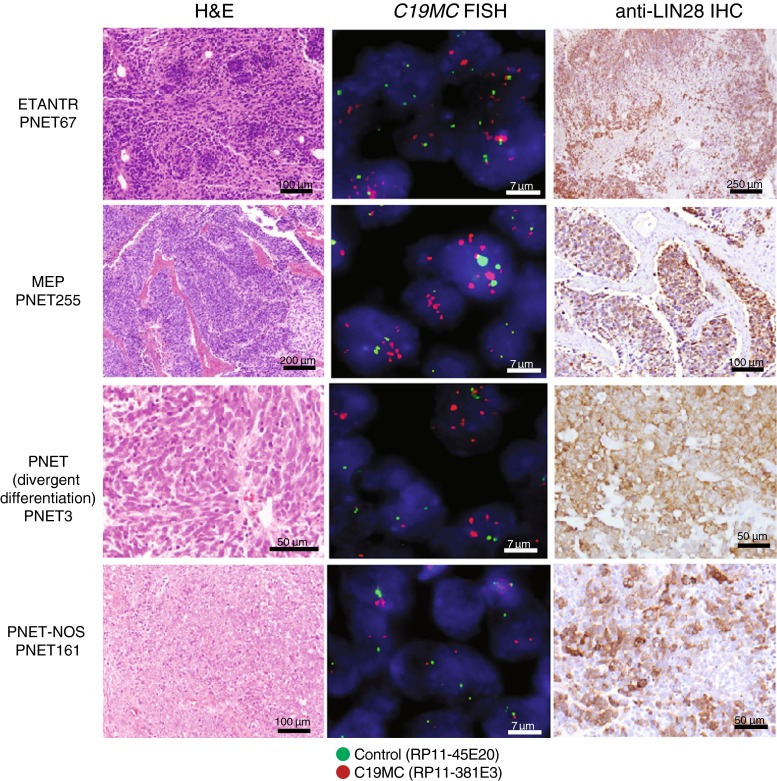
Histologic spectra of CNS-PNETs with *C19MC* amplification and LIN28 immunopositivity. Representative H and E stains, *C19MC* FISH and LIN28 IHC analyses of CNS-PNETs with histologic features of ETANTR (PNET67), MEP (PNET255), CNS-PNET with divergent differentiation (PNET3) and undifferentiated PNET-NOS (PNET161)

To evaluate the relationship of CNS-PNETs with *C19MC* amplification and LIN28 positivity to known histologic sub-types of CNS-PNETs [14], we next conducted more detailed evaluation of tumor histopathology and clinical features in an expanded cohort of 54 CNS-PNETs identified on the basis of *C19MC* amplification and/or LIN28 expression (Table 1, Supplemental Table 2). Interestingly, of 45 tumors with complete histopathologic review, 22/45 (49 %) exhibited ependymoblastic rosettes with neuropil consistent with a histologic diagnosis of ETANTR (including 3 tumors initially diagnosed as EPBs), 12/45 (26.7 %) displayed papillary features characteristic of MEP with variable amounts of neuropil and rosette formation, while a subset of 11/45 (24 %) tumors lacked characteristic histologic features of ETANTRs, EPBs or MEPs but exhibited variable differentiation or relatively bland histology compatible with classic or CNS-PNET-NOS (Fig. 1). In addition to spanning histologic sub-classes, we observed that *C19MC* amplified/LIN28+ CNS-PNETs also exhibited multiple CNS locations; cerebral origins were most common (41/54; 76 %), but 24 % (13/54) arose in other CNS locations including the cerebellum (6/54; 11 %), brain stem (5/54; 9 %), pre-sacral space (1/54; 2 %) and optic nerve (1/54; 2 %). Of note, 5/37 (14 %) tumors with overlapping data on *C19MC* and LIN28 status exhibited LIN28 positivity only, without changes in *C19MC* expression or copy number; these tumors were found in each histologic class (please see Supplementary Table 2 for details).

**Fig. 2:**
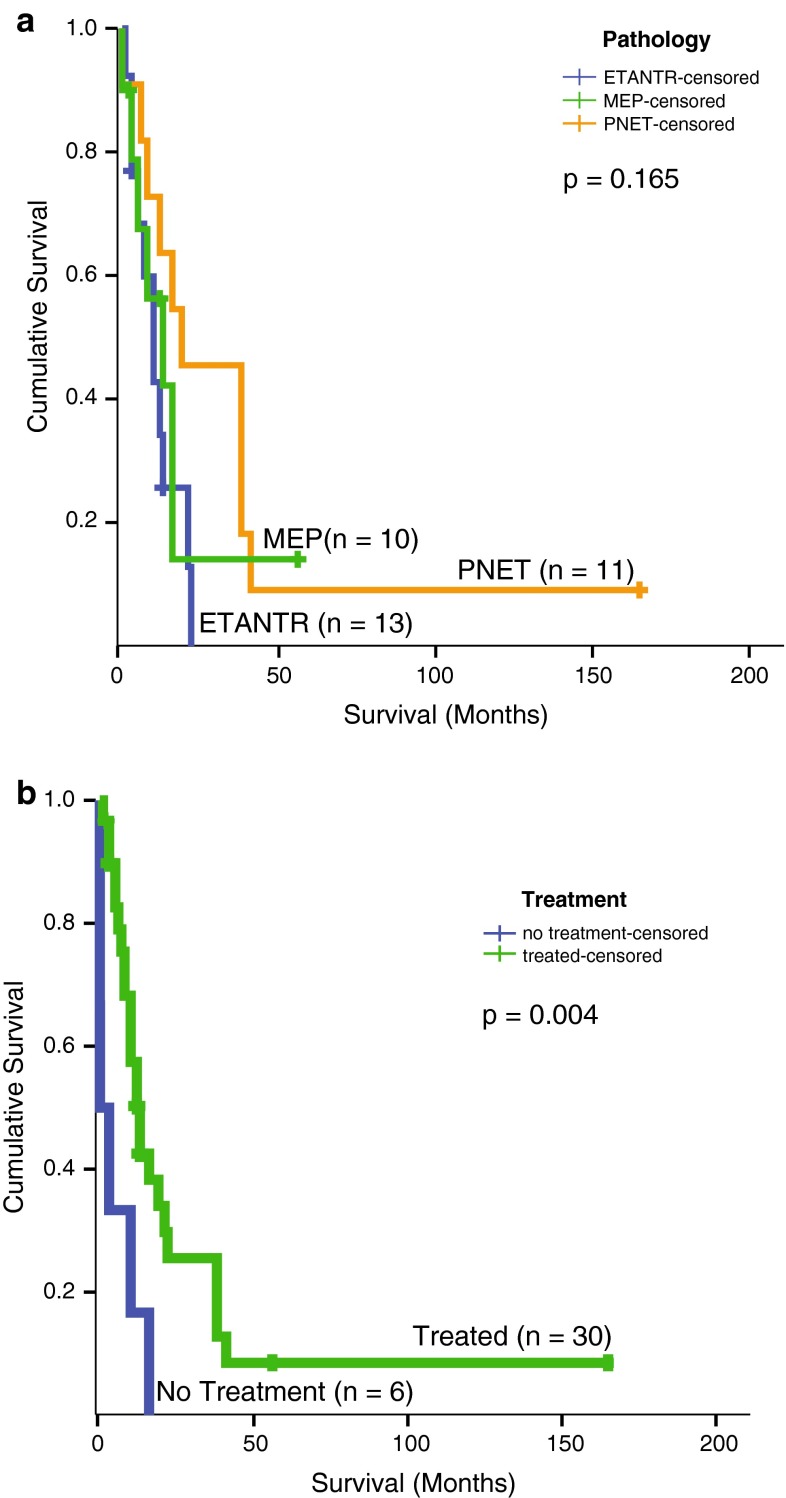
Survival analyses of *C19MC* amplified and/or LIN28+ CNS-PNETs. **a** Log-rank survival analysis stratified by histologic classes of CNS-PNETs; log-rank test comparing overall survival of ETANTR vs MEP vs PNET is shown. **b** Log-rank comparison of overall survival for all CNS-PNETs stratified by receipt of treatment versus no treatment

Analyses of clinical features showed that 24/37 (65 %) of tumors were non-metastatic at diagnosis and arose predominantly in young children with a median age of 29 months at diagnosis. The incidence of tumor metastases, patient demographics (gender and age) and overall survival did not differ significantly across histologic classes. Detailed survival data available for 36/54 patients indicated a similar highly aggressive course with rapid disease progression and death for a majority of patients regardless of tumor histologic class (Fig. 2a). The median survival for all patients was only 12 ± 1.9 months (range of 0–165 months). However, patients who received treatment had significantly longer survival as compared to untreated patients (median survival of 13 ± 1.9 versus 0.060 ± 1.84 months; Fig. 2b). Notably, six patients (5 with specific treatment information) had substantially longer survival of 38–204 months; three patients remained alive at 56, 165 and 204 months (Supplemental Table 2). These findings suggest that chemotherapy ± radiotherapy treatment may benefit a subset of patients.

### *C19MC* amplified/LIN28+ CNS-PNETs with various locations and histology exhibit common genetic and epigenetic signatures

**Fig. 3:**
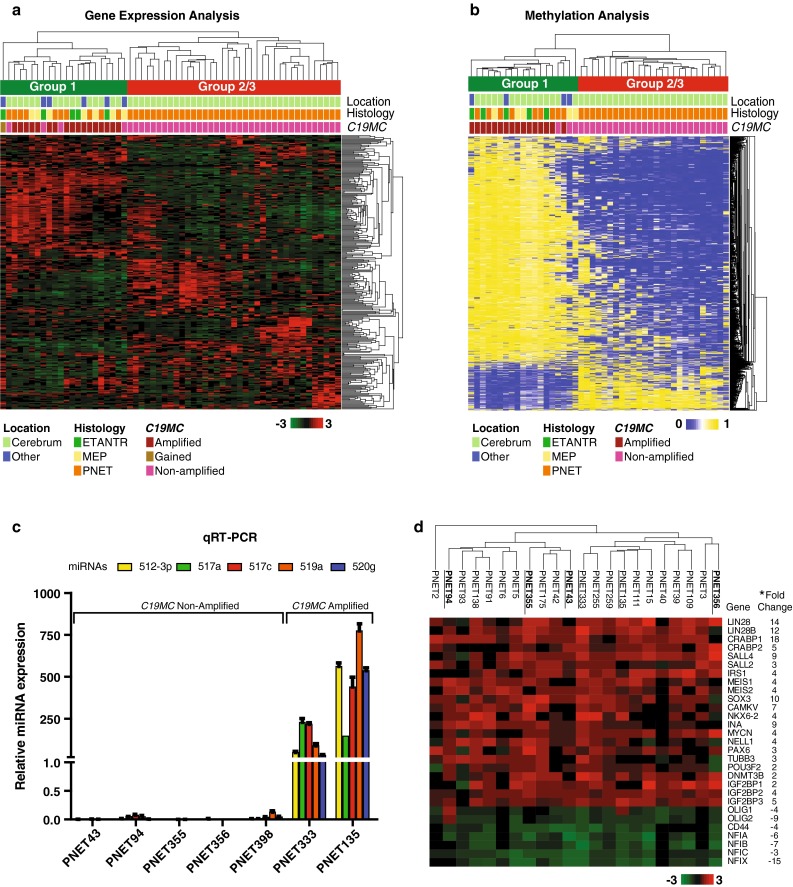
Unified gene expression and methylation signatures for *C19MC* amplified and/or LIN28 + CNS-PNETs. **a**, **b** Unsupervised hierarchical cluster analysis was performed on gene expression (**a**) and methylation data (**b**) generated, respectively, from 59 (Illumina HT-12v4 arrays) and 45 (Illumina human 450 k arrays) primary CNS-PNETs. Most stable tumor cluster patterns were achieved with a minimal set of 300 genes, and across 500–4000 methylation probes (Supplemental Fig. 2) and indicated distinct segregation of *C19MC* amplified/LIN8+ tumors from Groups 2/3 CNS-PNETs, which lack either feature. Anatomic location of individual tumors, specific CNS-PNET histology and *C19MC* genomic status are indicated. **c** Quantitative RT-PCR analyses of *C19MC* miRNAs: miR-512-3p, 517a, 517c, 519a, 520g, in a subset of CNS-PNETs without *C19MC* genomic amplification is shown relative to that of *C19MC* amplified tumors, RNU6B served as control. **d** Genes most highly enriched in group 1 versus group 2/3 CNS-PNETs were identified using a supervised *t* test adjusted for multiple hypothesis testing (false discovery rate ≤0.05). Heat map shows relative magnitude of enrichment for specific genes with functions in cell lineage, signaling and epigenomic regulation at a significance of *q ≤ 0.05. Tumor analyzed by qRT-PCR in **c** are *underlined*, non-*C19MC* amplified tumors and the *C19MC* amplified control tumor analyzed are, respectively, shown in *bold*

We had previously demonstrated using global gene expression profiles that *C19MC* amplified/LIN28+ CNS-PNETs arising in the cerebral hemispheres comprised a molecular class with relative enrichment of primitive neural features that was distinct from two other molecular groups (designated group 2/3) of hemispheric CNS-PNETs [17]. The convergence of clinical phenotypes observed in *C19MC* amplified/LIN28+ tumors with different histologic features and anatomic locations, prompted us to investigate whether all *C19MC* amplified/LIN28+ tumors, regardless of histology or location, comprised a common molecular entity. Indeed, unsupervised hierarchical cluster analyses of gene expression data from 22 *C19MC* amplified/LIN28+ tumors, which included five ETANTRs, seven MEPs, and ten PNETs from different CNS locations, clustered together as a common molecular class distinct from Group 2/3 CNS-PNETs (Fig. 3a). Similarly, unsupervised cluster analyses of global methylation profiles generated from an overlapping cohort of 19 *C19MC* amplified/LIN28+ tumors, comprised of 5 ETANTRs, 5 MEPs, and 9 PNETs from various locations, showed they formed a common molecular cluster distinct from the group 2/3 hemispheric CNS-PNETs (Fig. 3b). In keeping with our prior studies of hemispheric CNS-PNETs [13, 17], *C19MC* amplified/LIN28+ tumors with different location and histology also exhibited a gene expression signature enriched for pluripotent and neural cell lineage genes. Notably, all tumors with or without *C19MC* amplification expressed high levels of pluripotent genes LIN28/LIN28B, and low levels of neural differentiation genes including the NF1 family of transcription factors (Fig. 3c, d). Collectively, our analyses indicate that all CNS-PNETs with *C19MC* amplification and/or LIN28 immunopositivity, regardless of histologic sub-class or location, share similar molecular and genetic makeup, thus indicating they comprise a common biological and diagnostic entity.

### Candidate therapeutics for *C19MC* amplified and/or LIN28+ CNS-PNETs

**Fig. 4:**
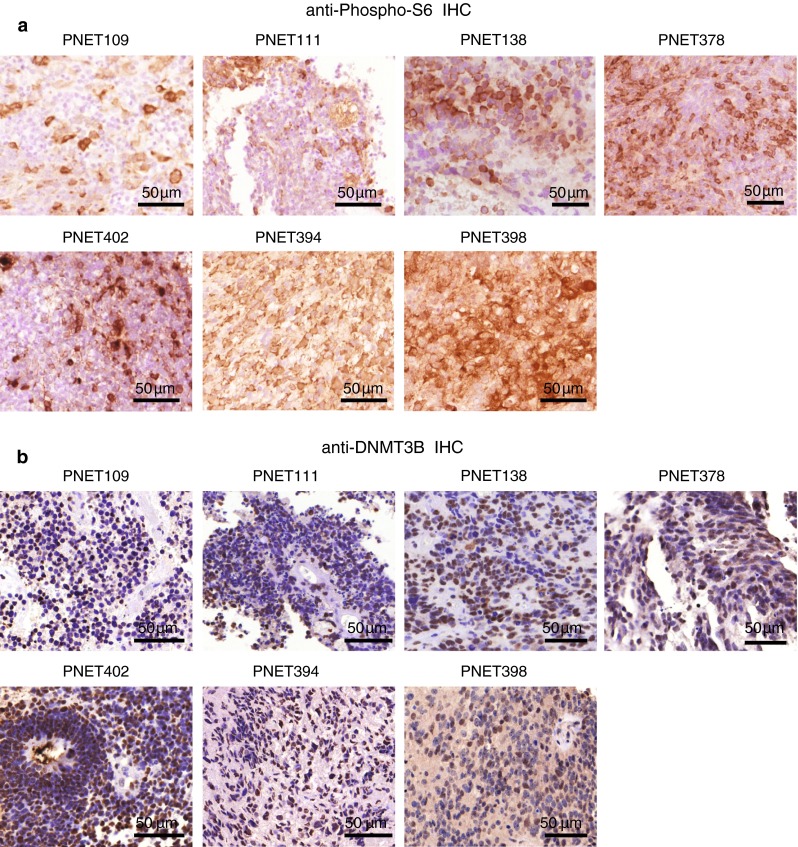
Expression of mTOR target, phospho-S6 and DNA methyl transferase, DNMT3B, in group 1 CNS-PNETs. Representative **a** phospho-S6 and **b** nuclear DNMT3B immunostain patterns in LIN28+ *C19MC* amplified (PNET 109, 111, 138, 378, 402) and non-amplified primary group 1 CNS-PNETs (PNET394 and 398)

In recent studies, we showed that the LIN28/let7/P13K-mTOR pathway is up-regulated and can be targeted by Rapamycin in a *C19M*C amplified primary ETANTR cell line [19]. In addition, we demonstrated that the unique methylation landscape of *C19MC* amplified tumors is in part imposed via *C19MC*-mediated repression of *RBL2*, and consequent up-regulation of DNMT3B [9]. The unified molecular signature of LIN28+/C19*MC* amplified and non-amplified CNS-PNET indicated that they may share common therapeutic pathways. We observed that LIN28/LIN28B and P13K-mTOR components, IGFBP1-3, which have been linked to a common functional pathway [20, 22], as well as DNMT3B were enriched in gene expression signatures of CNS-PNETs with or without *C19MC* amplification (Fig. 3d). Consistent with these observations, IHC analyses revealed up-regulation of phospho-S6 (pS6), an mTOR pathway target, as well as DNMT3B in *C19MC* amplified and non-amplified primary tumors (Fig. 4a, b).

**Fig. 5:**
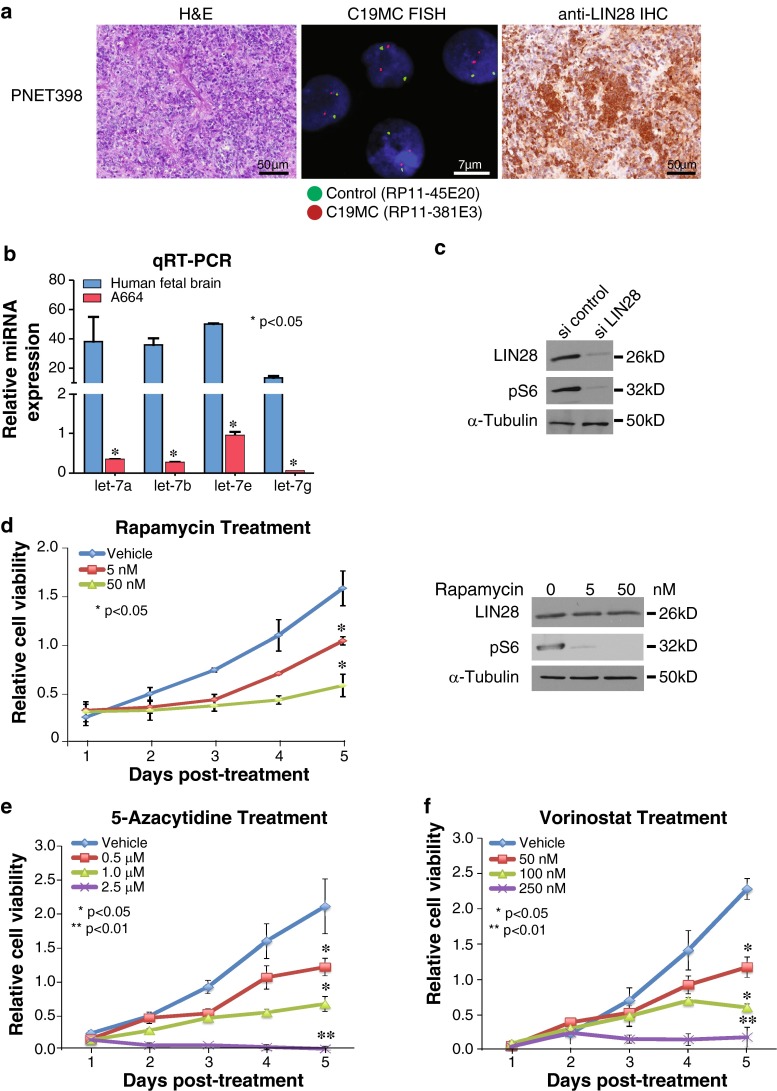
Treatment of group 1 CNS-PNET cells with rapamycin, 5-azacytidine and vorinostat. A stable cell line from a primary non-*C19MC* amplified/LIN28+ CNS-PNET was established and tested for sensitivity to inhibitors of mTOR signaling and epigenomic modifiers as described in methods. **a** H and E stains, *C19MC* FISH and LIN28 IHC analysis of primary PNET398 from which the A664 cell line was derived, indicating lack of *C19MC* amplification and strong LIN28 immunopositivity. ** b**–**c** Quantitative RT-PCR and Western blot analyses indicating an intact LIN28-let7/mTOR axis in A664 cells. Expression of let-7a, 7b, 7e and 7g miRNAs was determined relative to that in normal human 16-week-old fetal brain and normalized to RNU6B. Results are shown as mean ± SEM; *n* = 2. A664 cells were treated with scrambled, control siRNA and siRNA directed against LIN28 and examined for expression of LIN28 and pS6 with α-tubulin as loading control. **d**–**f** A664 cells were treated with varying doses of rapamycin, 5-azacytidine and vorinostat, and drug effect on cell viability was measured using MTS assays. Plots represent summary of three independent experiments with three replicas/data point; *error bars* represent SEM. *p ≤ 0.05. *Right panel* in figure **d** shows Western blot analyses for LIN28 and pS6 expression in rapamycin-treated A664 cells; tubulin served as loading control

To investigate whether pharmacologic inhibitors of mTOR and DNA methylation may have therapeutic roles in all group 1 CNS-PNETs, we tested the effects of rapamycin as well pharmacologic inhibitors of DNA methyl transferase function (5-azacytidine and vorinostat), on growth of a tumor cell line (A664) derived from a non-*C19MC* amplified primary cerebral PNET398. Using quantitative RT-PCR and siRNA-mediated knockdown of LIN28, we confirmed that the LIN28/let7/P13K-mTOR axis was conserved in A664 cells. Indeed, rapamycin treatment significantly inhibited A664 cell growth with concomitant down-regulation of pS6 expression (Fig. 5b–d). Similarly, we observed that 5-azacytidine and vorinostat significantly inhibited A664 cell growth in a dose-dependent manner (Fig. 5e, f). Collectively, these data highlight regulators of P13K/mTOR signaling, as well epigenomic modifiers, as novel promising therapeutic targets for these recalcitrant infantile tumors.

## Discussion

In prior studies, we identified three transcriptional classes of CNS-PNETs arising in the cerebral hemispheres. Specifically, we reported that amplification of the oncogenic *C19MC* miRNA locus and/or high expression of LIN28, a pluripotency gene, identified a distinctively aggressive sub-group of hemispheric tumors, which we called group 1 tumors [17]. In this study, we demonstrate that *C19MC* amplification and/or LIN28 expression are seen in CNS-PNETs with a spectrum of histology and location, and overlapping transcriptional and epigenomic signatures that are distinct from that of other molecular sub-types of CNS-PNETs arising in the cerebral hemispheres [13, 17]. Specifically, our data suggest that current known histologic categories of CNS-PNETs which include ETANTRs, MEPs and EPBs which arise in different CNS locations comprise common molecular and therapeutic entities.

Our analyses of a large cohort and spectrum of malignant pediatric brain tumors indicate that *C19MC* amplification is exclusively associated with group 1 CNS-PNETs. Specifically, we did not observe high-level DNA copy number changes of *C19MC* in any other malignant pediatric brain tumors with confirmed histopathologic diagnostic features of MBs, ATRTs, EPNs, HGGs, and CPCs. The pathogenic and diagnostic importance of this locus in CNS-PNETs is further highlighted by recent identification of *TTYH1*:*C19MC* gene fusions which is associated with very high expression of specific *C19MC* miRNAs and suggest *C19MC* drives oncogenesis in part by facilitating maintenance and transformation of a very early, neural compartment [9]. Notably, although we observed cytoplasmic LIN28 expression uniformly (100 %) in *C19MC* amplified CNS-PNETs, our analyses also revealed cytoplasmic as well as nuclear LIN28 staining in a subset of MBs, ATRTs, EPNs and HGGs but not CPCs. Similar to prior reports of cytoplasmic LIN28 staining in 20–60 % of pediatric and adult gliomas [15] and 64 % of ATRTs [3], we observed strong LIN28 cytoplasmic staining in up to 20–25 % of ATRTs and HGGs analyzed (Supplemental Tables 1, 3), which contrasts with a report of cytoplasmic LIN28 immunostaining exclusively in ETANTRs [11]. The reason underlying these discrepant observations is unclear and may be related to the limitations of tissue microarray analyses to comprehensively capture tumor heterogeneity. Thus we propose that a combination of tumor morphology, together with cytoplasmic LIN28 immunostaining and *C19MC* genetic status, is needed to robustly distinguish group 1 CNS-PNETs or related embryonal tumors from other malignant pediatric brain tumors which may exhibit varying LIN28 expression.

Notably, as in our prior study [17], we observed that *C19MC* amplification or copy number gains together with high LIN28 expression identified CNS-PNETs that exhibited predominantly primitive neural histology with varying proportions of ependymoblastic rosettes, neural epithelium in papillary and pseudo-tubular formation, although such features were not necessarily identifiable in all *C19MC* amplified/LIN28+ CNS-PNET samples examined. We also observed that the proportion of cells with *C19MC* amplification and/or LIN28 expression varied across tumor samples that share group 1 CNS-PNETs molecular signatures, thus indicating that histopathologic analyses may be confounded by intra-tumoral heterogeneity which may reflect a varying, continuum of differentiation in the tumors. Indeed this may also apply to the histopathologic spectrum of ETANTRs, EPBs and MEPs seen in CNS-PNETs [7]. Although each of these entities are reported to be extremely rare, our data suggest that a combination of *C19MC* amplification and/or LIN28 expression together with careful morphologic assessment of tumor may identify up to 25 % of CNS-PNETs that make up this histogenetic tumor spectrum. As these tumors predominantly arise in children <4 years of age, they may represent an even higher proportion of brain and other CNS tumors diagnosed in infancy. Thus, a diagnostic approach which combines histopathologic assessment together with evaluation of *C19MC* and LIN28 status will be important for capturing the true spectra of this disease. Therefore, we suggest that evaluation of *C19MC* and LIN28 status should be considered for all malignant neuroepithelial tumors arising in young children, regardless of CNS locations, in a manner similar to the diagnostic work-up for ATRTs.

We did not observe significant differences in *C19MC* amplification and LIN28 expression status nor in global identifiers based on gene expression and methylation analyses between various histological sub-types of CNS-PNETs. In addition, though trends toward older age and lower incidence of metastasis at diagnosis were observed in tumors with a PNET or variant PNET diagnosis, no significant differences were evident between histological subgroups. These subtle distinctions may prove to be clinically relevant upon analysis of a larger tumor cohort. Our data suggest that the majority of these tumors are localized at presentation, however, interestingly a subset of patients exhibited unusual patterns of metastasis including dural invasion and spread to extra-neural sites (Supplemental Table 2). Comprehensive diagnostic evaluation of large unbiased cohorts will be important for revealing disease patterns to inform and unify diagnostic work-up and therapeutic approaches for these rare tumors.

Consistent with prior studies [11, 17], we observed dismal overall survival in our study cohort with only 3/36 patients who remain alive 56–204 months after diagnosis. Our data suggest a survival benefit for a small proportion of patients treated with chemotherapeutic regimes, with or without radiotherapy and underscore the need for better therapies in this disease. Our recent [19] and current study which demonstrates that rapamycin, a PI3K/mTOR inhibitor, significantly inhibits growth of cell lines derived from both *C19MC* amplified and non-amplified primary group 1 tumors suggests targeting the P13K/mTOR pathway as a novel therapeutic avenue for this disease. In this study, we also demonstrated that 5-azacytidine, a pharmacological antagonist of DNMTs, and HDAC inhibitor, vorinostat, had significant effects on A664 cell growth. Together with recent demonstration that *C19MC* regulates the RBL2-DNMT3B axis [9], our data suggest epigenetic regulators as important new therapeutic targets in this disease.

 In summary, our study, together with a recent similar report by Korshunov et al. [12], indicate CNS-PNETs with *C19MC* amplification and/or LIN28 expression span various histologies but comprise a single molecular disease that warrant common therapeutic strategies. Our study provide novel insights into potential targetable pathways for this frequently fatal but relatively uncommon disease and report on a unique cell line model that will be an invaluable resource for future therapeutic investigations.

## Electronic supplementary material

Below is the link to the electronic supplementary material.

Figure 1 Representative patterns of LIN28 immuno-stains in a spectrum of malignant primary pediatric brain tumors. Immunohistochemical analyses were performed to assess LIN28 expression in primary pediatric tumors with histologic diagnosis of MBs, EPNs, HGGs and ATRTs. Quantitative and qualitative assessment of LIN28 expression were determined as described in methods using a numerical score for intensity and distribution of stains, and nuclear versus cytoplasmic expression (Supplemental Table 3). Representative images for cytoplasmic and nuclear staining patterns for LIN28 in each histologic category of tumor is shown in Figure a. Representative BAF47 immuno-stains, used to confirm diagnostic loss of SMARCB1 protein expression in ATRTs, is shown in Figure b. Supplementary material 1 (EPS 228985 kb)

Figure 2 NMF consensus cluster analyses of methylation profiles in CNS-PNETs. Methylation data generated from 45 primary CNS-PNETs (Illumina Infinium 450K Human Methylation arrays) were analyzed using NMF (Non-negative matrix factorization) consensus cluster methods. Cluster patterns were determined re-iteratively using 200 – 10,000 probes ranked by standard deviation to establish the most stable tumor clusters achievable with a minimal gene set. Highest cophenetic coefficient was achieved with 500-4,000 probes. NMF analyses plots of a minimal 500 probe set (red box) which revealed the most stable cluster patterns with highest cophenetic coefficient at k=2 and corresponding heat map are shown in red boxes. Supplementary material 2 (EPS 1970 kb)

Figure 3 Histopathologic diagnostic features of a medulloepithelioma. A representative H and E stain of a medulloepithelioma showing characteristic histopathologic diagnostic features which include presence of papillary, tubular or trabecular arrangements of neoplastic neuro-epithelium resembling an embryonic neural tube. Supplementary material 3 (EPS 13654 kb)

Supplemental Table 1 *C19MC* amplification and LIN28 expression in various primary pediatric brain tumors. Supplementary material 4 (XLSX 12 kb)

Supplemental Table 2 Clinico-pathologic and molecular features of *C19MC* amplified/LIN28+ CNS-PNETs. Supplementary material 5 (XLSX 19 kb)

Supplemental Table 3 Detailed analyses for *C19MC* amplification and LIN28 expression in various pediatric brain tumors. Supplementary material 6 (XLS 193 kb)
